# Clinical effectiveness of hypertonic sodium lactate infusion for intraoperative brain relaxation in patients undergoing scheduled craniotomy for supratentorial brain tumor resection: A study protocol of a single center double-blind randomized controlled phase II pilot trial

**DOI:** 10.1097/MD.0000000000031038

**Published:** 2022-10-07

**Authors:** Guillaume Besch, Anne-Laure Parmentier, Francis Berthier, Hélène Jaeg, Julien Villeneuve, Fethi Hammoudi, Nans Scaringella, Anne-Laure Clairet, Lucie Vettoretti, Gilles Chopard, Laurent Thines, David Ferreira, Emmanuel Samain, Sebastien Pili-Floury

**Affiliations:** a Department of Anesthesiology and Intensive Care Medicine, University Hospital of Besancon, and EA 3920, University of Franche-Comte, Besancon, France; b Clinical Methodology Center, INSERM CIC 1431, University Hospital of Besancon, and UMR 6249 Chrono Environment, University of Franche-Comte, Besancon, France; c Department of Anesthesiology and Intensive Care Medicine, University Hospital of Besancon, Besancon, France; d Department of Pharmacy, University Hospital of Besancon, and Interaction Hôte-Greffon-Tumeur/Ingénierie Cellulaire et Génique, University of Bourgogne Franche-Comte (UBFC), INSERM, EFS BFC, Besancon, France; e Department of Neurology, University Hospital of Besancon, and EA 481 Neuroscience, IFR 133, University of Bourgogne Franche-Comte, Besancon, France; f Department of Neurosurgery, University Hospital of Besancon, Besancon, France; g Department of Anesthesiology and Intensive Care Medicine, University Hospital of Besancon, and EA 481 Neuroscience, University of Bourgogne Franche-Comte, Besancon, France.

**Keywords:** anesthesia, brain relaxation, craniotomy, intracranial pressure, mannitol, neurosurgery, sodium lactate

## Abstract

Hyperosmolar solutions are prescribed in neurosurgery patients to provide satisfactory intraoperative brain relaxation and to lower cerebral injuries related to surgical retractors. Mannitol is traditionally considered as the first-choice solution for brain relaxation in neurosurgery patients. Hypertonic sodium lactate infusion was reported to provide a higher and longer osmotic effect compared to mannitol in severely brain-injured patients and to prevent impaired cerebral energetics related to brain injuries. To date, the clinical effectiveness of hypertonic sodium lactate infusion has never been studied in neurosurgery patients. The hypothesis of the study is that hyperosmolar sodium lactate infusion may provide satisfactory intraoperative brain relaxation in patients undergoing scheduled craniotomy for supratentorial brain tumor resection.

**Methods and analysis:**

We designed a phase II randomized, controlled, double-blind, single-center pilot trial, and aim to include 50 adult patients scheduled for craniotomy for supratentorial brain tumor resection under general anesthesia. Patients will be randomized to receive either mannitol (conventional group) or hypertonic sodium lactate (intervention group) infusion at the time of skin incision. Brain relaxation (primary outcome) will be assessed immediately after opening the dura by the neurosurgeon blinded to the treatment allocated using a validated 4-point scale. The primary outcome is the proportion of satisfactory brain relaxation, defined as brain relaxation score of 3 or 4.

**Ethics and dissemination:**

This study was approved by the Ethics Committee (Comité de Protection des Personnes Est III) and authorized by the French Health Authority (Agence Nationale de Sécurité des Médicaments, Saint-Denis, France). The University Hospital of Besancon is the trial sponsor and the holder of all data and publication rights. Results of the study will be submitted for publication in a peer-review international medical journal and for presentation in abstract (oral or poster) in international peer-reviewed congresses.

**Registration:**

The trial is registered with ClinicalTrials.gov (Identifier: NCT04488874, principal investigator: Prof Guillaume Besch, date of registration: July 28, 2020).

**Key Points**

- This is the first study to investigate the clinical effectiveness of molar sodium lactate infusion for intraoperative brain relaxation in patients scheduled for craniotomy.
- Brain relaxation is assessed using a validated 4-point scale by the neurosurgeon in charge of the patient blinded to the treatment allocated.
- Single-center double-blind randomized phase II pilot trial.
- The study is not designed to compare the 2 groups with adequate statistical power but will provide high-quality data to design further studies.

## 1. Introduction

The management of cerebral edema and intracranial pressure (ICP) is a critical component of intraoperative care of patients undergoing brain surgery.^[1,2]^ Tumor size and peritumoral brain edema increase ICP and satisfactory brain relaxation is crucial to improve surgical conditions in patients undergoing craniotomy for supratentorial brain tumor resection.^[1]^ Surgical retractors used to access the surgical site locally increase the pressure exerted on the underlying parenchyma and can contribute to retraction ischemia reperfusion injuries and vasogenic edema in healthy brain areas.^[3,4]^

Hyperosmolar solutions are widely prescribed in neurosurgery patients to decrease ICP and to improve brain relaxation prior to surgical incision.^[1]^ The hyperosmolarity combined to impermeability of the blood–brain barrier to osmotically active substances favor the move of water from the brain to the intravascular compartment.^[1]^ Mannitol is traditionally considered the standard and the first-choice solution for brain relaxation in neurosurgery patients.^[1]^ The mannitol effect is mainly related to an osmotic mechanism leading to brain dehydration and brain relaxation. Moreover, mannitol could increase cerebrospinal fluid absorption and cerebral perfusion pressure leading to vasoconstriction and decreased cerebral blood volume.^[5–7]^ The dose of 1.0 g/kg of 20% mannitol infused at the time of skin incision appeared to have the best benefits risks ratio in patients undergoing craniotomy for supratentorial brain tumor.^[8]^ However, it provides satisfactory brain relaxation in only 65% of patients and side effects such as dehydration, hypotension, renal failure, and rebound effect after transitory action was reported.^[8–10]^

Molar sodium lactate was proposed as an alternative to 20% mannitol in severe brain-injured patients.^[11,12]^ Two randomized trials reported that hypertonic sodium lactate infusion might be more efficient than 20% mannitol to prevent and treat ICP episodes after traumatic brain injury.^[11,12]^ The reduction in ICP provided by hypertonic sodium lactate could stay longer since lactate catabolism triggers a shift of chloride anion from the intracellular to the extracellular compartment to maintain plasma electroneutrality.^[13]^ The shift of chloride anion could lead to a reduction in brain volume resulting from the movement of water from the brain to the intravascular comportment that persists several hours after the end of lactate sodium infusion without any rebound effect.^[14]^ Moreover, sodium lactate infusion might contribute to increase intracerebral concentration of lactate that could prevent the impaired cerebral energetics related to brain ischemia reperfusion injuries.^[15–19]^ To date, the clinical effectiveness and safety of hypertonic sodium lactate infusion for brain relaxation in patients undergoing scheduled craniotomy for supratentorial brain tumor resection have never been investigated.

## 2. Objectives

The study was designed to describe the clinical effectiveness of hypertonic sodium lactate infusion on intraoperative brain relaxation in patients undergoing scheduled craniotomy for supratentorial brain tumor resection.

## 3. Methods and Analysis

This study is written in accordance with the SPIRIT guidelines for the reporting of interventional trial protocols.^[20]^

### 3.1. Trial design

This study is a phase II prospective randomized, controlled, double-blind, single center pilot trial.

### 3.2. Eligibility

Adult patients scheduled for craniotomy for supratentorial brain tumor resection under general anesthesia in the Ne University Hospital of Besancon, France (Centre Hospitalier Universitaire (CHU) de Besancon) are eligible for inclusion. Inclusion and exclusion criteria for the present study are listed below.

### 3.3. Inclusion criteria

- Signed informed consent.
- American Society of Anesthesiologists’ (ASA) physical status I to III.
- Scheduled craniotomy for supratentorial brain tumor resection under general anesthesia requiring brain relaxation.
- Unilateral brain tumor.
- Ability to speak, write, and understand French language.
- Patient affiliated to French Social Security or equivalent.

### 3.4. Exclusion criteria

- ASA physical status IV or higher.
- Emergent surgery.
- Age under 18 years or over 75 years.
- Preoperative Glasgow Coma Scale < 13.
- Body mass index < 18 kg/m^2^ or > 30 kg/m^2^.
- Body weight > 100 kg.
- Hyponatremia < 130 mmol/L or hypernatremia > 145 mmol/L.
- Hypokalemia < 3.5 mmol/L.
- Preoperative osmotherapy (mannitol, hypertonic saline, or hypertonic sodium lactate) within 24 h prior surgery.
- Congestive heart failure.
- Estimated glomerular filtration rate (MDRD formula) < 60 ml/min/1.73 m^2^.
- Moderate to severe liver dysfunction with a Child Pugh ≥ B7.
- Myasthenia.
- Extra ventricular drainage or ventriculoperitoneal shunt.
- Diagnosed dementia.
- Contraindication to propofol, remifentanil, atracurium, or cisatracurium administration.
- Allergy to mannitol or any of its excipients.
- Allergy to hypertonic sodium lactate or any of its excipients.
- Legal incapacity or limited legal capacity.
- Subjects without health insurance.
- Pregnant of breast-feeding women.
- Subjects within the exclusion period of another study.

### 3.5. Study outline

Eligible patients are screened and informed during the anesthesia consultation. Written informed consent is obtained the day before surgery by investigators after oral explanation of the study. After written informed consent is obtained, patients are randomized using a computer-generated randomization list implemented on the CleanWeb web-based system (Telemedicine Technologies, France).

After randomization, a first cognitive assessment using a battery of validated neuropsychological tests (see below) is performed by an investigator who was specifically trained by a neuropsychologist (GC) prior to the start of the study. Patient characteristics, past medical history including ASA physical status, type of brain tumor, preoperative management of brain tumor (corticosteroid therapy, antiepileptic therapy, chemotherapy, and/or radiotherapy), preoperative Karnosky performance status, and preoperative neurological status including Glasgow Coma Scale are collected. Midline shift, brain tumor size, and localization, and the size of peritumoral edema are extracted from the preoperative brain imaging [either brain magnetic resonance imaging (MRI) or computed tomography scan]. Included patients undergo a first cognitive assessment using a battery of validated neuropsychological tests (see below).

On arrival in the operating room, standard monitoring (General Electric Healthcare) is put in place. A peripheral venous catheter dedicated to anesthetic drug infusion is inserted in a forearm vein. All patients receive intravenous anesthesia based on manually driven target-controlled infusion of propofol and remifentanil aiming to maintain bispectral index (BIS) values between 40 and 60. All patients are paralyzed using nondepolarizing neuromuscular blocking agent (either cisatracurium or atracurium).

After induction of anesthesia, patients are intubated, and tidal volume and respiratory rate are set to maintain the end-tidal carbon dioxide between 35 and 40 mm Hg. Inspired oxygen fraction is adjusted to obtain a pulse oximetry ≥ 95%. A second peripheral venous catheter, a urinary catheter, and an indwelling arterial catheter introduced in a radial artery are implemented.

The treatment allocated by randomization for brain relaxation, that is, mannitol (conventional group) or hypertonic sodium lactate (intervention group) infusion, is started at the time of skin incision and is administered over 15 to 20 minutes (see below). The degree of brain relaxation (primary outcome) is assessed by the neurosurgeon immediately after opening the dura. If a greater degree of brain relaxation is required, a rescue therapy is administered according to the following stepwise strategy: (1) administration of additional mannitol 0.25 g/kg; (2) deepening anesthesia to achieve a BIS value between 20 and 40; and (3) administration of an intravenous bolus of sodium thiopental 5 mg/kg.

Blood samples are collected immediately before skin incision (baseline, T_0_), and at 30 minutes, 60 minutes, 180 minutes, 24 hours, and 48 hours after the end of osmotherapy (either mannitol or hypertonic sodium lactate) to measure blood pH, natremia, kaliemia, lactatemia, and serum osmolality. Serum levels of S100B protein and neuron-specific enolase (NSE) are measured at baseline, and at 24 hours and 48 hours after the end of osmotherapy.

The following surgical and anesthetic details are recorded: type and duration of surgery; intraoperative blood loss and urine output, blood transfusion and total volume of fluids infused; type and total dose of drugs administered; and all blood pressure, heart rate, pulse oximetry, and end-tidal carbon dioxide values from induction of anesthesia to discharge from the operating room.

At the end of surgery, patients are admitted to the postoperative surgical intensive care unit during at least the first postoperative 24 hours. Postoperative neurological status (Glasgow Coma Scale, new transient, or permanent neurological deficits), urine output, blood transfusion, and total volume of fluids infused are assessed on days 1 and 2 after surgery. Brain MRI is performed within the first 2 days after surgery to quantify postoperative brain edema and ischemic changes, and postoperative midline shift.

Karnosky performance status, postoperative morbidity (cardiac arrythmia within 2 days after surgery, length of stay in the intensive care unit and in the hospital, surgical wound infection, pneumonia, readmission in the intensive care unit or in the hospital, surgical revision for postoperative intracerebral hematoma and/or life-threatening intracranial hypertension, coma, seizure, stroke, any permanent neurological deficit, any drug-related adverse event), and postoperative mortality are assessed on day 30 after surgery.

### 3.6. Randomization, allocation concealment, and blinding

Randomization is performed the day before surgery using the CleanWeb web-based system (Telemedicine Technologies, France). A computer-generated permuted-block randomization list with varying block sizes (ratio 1:1) was implemented on the website at the beginning of the study by an independent data manager. The block size is unknown to the investigators. When written informed consent was obtained, the investigator entered data online regarding each patient to enable inclusion and randomization. The treatment randomly allocated to the patient is immediately provided to the investigator by the website.

Patients and outcome assessors (neurosurgeons) are blinded to the treatment allocated.

### 3.7. Study procedure and interventions

In the interventional group, patients receive hypertonic sodium lactate infusion for brain relaxation. The intravenous infusion of hypertonic sodium lactate (Na^+^ 1000 mmol/L, lactate 1000 mmol/L, prepared by the Etablissement Pharmaceutique Hopitaux de Paris, Assistance Publique Hopitaux de Paris, Paris) is started at the time of skin incision and is administered over 15 to 20 minutes at 2.5 mL/kg (maximal dose of 250 mL; patients whose weight is > 100 kg are excluded).

In the conventional group, patients receive an equimolar dose of 20% mannitol infusion for brain relaxation. The intravenous infusion of 20% mannitol is started at the time of skin incision and is administered over 15 to 20 minutes at 5 mL/kg (1 g/kg) (maximal dose of 500 mL; patients whose weight is > 100 kg are excluded). Seo et al reported that 1 g/kg was the optimal dose of mannitol for intraoperative brain relaxation in patients undergoing craniotomy for supratentorial brain tumor resection.^[8]^

If a greater degree of brain relaxation is required during surgery, a rescue therapy is administered according to the following stepwise strategy: (1) administration of additional mannitol 0.25 g/kg; (2) deepening anesthesia to achieve a BIS value between 20 and 40; and (3) administration of an intravenous bolus of sodium thiopental 5 mg/kg.

### 3.8. Outcome measures

The primary outcome is the proportion of satisfactory brain relaxation, defined as brain relaxation score of 3 or 4. Brain relaxation is assessed immediately after opening the dura by the neurosurgeon in charge of the patient blinded to the treatment allocated. The assessment of brain relaxation is performed using a validated 4-point scale,^[21]^ with: 1 = bulging brain; 2 = firm brain; 3 = satisfactorily relaxed; and 4 = perfectly relaxed).

Secondary outcomes are proportion of patients requiring a rescue therapy to improve the degree of brain relaxation during surgery; blood pH, serum osmolality, natremia, kaliemia, and lactatemia at baseline (before skin incision), and at 30 minutes, 60 minutes, 180 minutes, 24 hours, and 48 hours after the end of osmotherapy (either mannitol or hypertonic sodium lactate); serum levels of S100B and NSE proteins at baseline and at 24 hours and 48 hours after the end of osmotherapy; postoperative recovery assessed by the Glasgow Coma Scale on day 1 and on day 2 after surgery and by the Karnofsky performance status on day 30 after surgery; cognitive performance at baseline (the day before surgery) and on day 2 after surgery; volume of postoperative brain edema assessed on brain MRI performed on day 2 after surgery; and postoperative morbidity and mortality on day 30 after surgery.

Cognitive performance is assessed the day before surgery and on day 2 after surgery using a battery of validated neuropsychological tests during a 20-min face-to-face interview with an investigator who was specifically trained by a neuropsychologist (GC) prior the start of the study. Seven neuropsychological tests are performed to explore 5 different cognitive domains: (a) attention, using the Trail Making Test, part A; (b) executive function, using the Trail Making Test, part B and the semantic-phonemic switching verbal fluency test; (c) verbal fluency, using the Isaacs Set Test and the semantic-phonemic clustering verbal fluency test; (d) learning and episodic verbal memory, using the Memory Impairment Screen test; and (e) verbal short term and working memory, using the forward and reverse digit span memory test.

Postoperative morbidity includes cardiac arrythmia that occurs within 2 days after surgery, any complication requiring surgical revision, unexpected cerebral edema, intracranial hypertension, intracerebral hematoma, stroke, seizure, coma, readmission in the intensive care unit or in the hospital, surgical wound infection, or pneumonia.

### 3.9. Laboratory measurements

Blood pH, natremia, kaliemia, lactatemia, and serum osmolality are measured at baseline (before skin incision), and at 30 minutes, 60 minutes, 180 minutes, 24 hours, and 48 hours after the end of osmotherapy (either mannitol or hypertonic sodium lactate) to describe the effect of hypertonic sodium lactate infusion on acid–base balance, electrolytes, and osmolality.

Serum levels of S100B and NSE proteins are measured at baseline (the day before surgery) and at 24 hours and 48 hours after the end of osmotherapy to assess postoperative brain ischemic changes.

### 3.10. Safety

All serious adverse events are collected and reviewed by the principal investigator and reported to the trial sponsor (CHU Besancon, Besancon, France), to the Pharmacovigilance Department of our institution and to the independent Data Safety and Monitoring Board (DSMB). Study insurance has been contracted for all participants by the trial sponsor (CHU Besancon, Besancon, France).

Since off-labeled use of hypertonic sodium lactate is prescribed in patients included in the interventional group. All serious adverse events will be reviewed and analyzed by the independent DSMB after the inclusion of the first 25 patients to decide whether the study should be stopped prematurely for safety reasons.

### 3.11. Sample size calculation

No previous study has assessed the clinical effectiveness of hypertonic sodium lactate infusion for intraoperative brain relaxation in patients undergoing scheduled craniotomy for supratentorial brain tumor resection. Thus, no assumption can be made on the expected difference in primary outcome between groups. The sample size calculation was based on the expected measurement accuracy of the primary outcome in the interventional group.

Seo et al reported a satisfactory brain relaxation (primary outcome) in 65% of patients who received the same dose of 20% mannitol as in the conventional group.^[8]^ We hypothesized that the primary outcome will be at least 55% in the interventional group to consider that hypertonic lactate sodium infusion provides a clinically relevant brain relaxation for supratentorial brain tumor resection.

A sample size of 25 patients was calculated to achieve a measurement accuracy of the primary outcome of 20% in the interventional group. In this phase II pilot study, 2 groups of patients are included to check whether the expected rate of the primary outcome is the same in the conventional group as in the study from Seo et al^[8]^ and to provide reliable data for further studies. Thus, the inclusion of a total sample size of 50 patients (25 patients per group) is planned.

### 3.12. Statistical analysis

The primary outcome will be described in the interventional group using mean and the 95% confidence interval of the mean. The normality of the distribution of continuous variable will be tested using the Shapiro–Wilk test. The interventional and conventional groups will be compared based on an intention-to-treat analysis. Intergroup comparisons will be conducted using the Mann–Whitney *U* or Student *t* tests for quantitative variables, depending on the distribution of data, and using Fisher’s exact test or the Chi-square test for qualitative variables. The significance level is fixed at 0.05. No interim analysis is planned.

### 3.13. Monitoring

The Department of Research and Clinical Investigation of our institution will monitor all written informed consent, inclusion and exclusion criteria, and check all serious adverse events.

## 4. Ethics and dissemination

### 4.1. Ethics approval and registration

This study was approved by the French Ethics Committee (Comité de Protection des Personnes Est III, Hopital de Brabois, CHU de Nancy, Chairperson Dr Patrick Peton, no. 18.11.19 on December 4, 2018) and authorized by the French Health Authority (Agence Nationale de Sécurité du Médicament, no. MEDAECPP-2018-10-00003 on December 11, 2018). The study is registered on ClinicalTrials.gov (Identifier: NCT04488874, principal investigator: Prof Guillaume Besch, date of registration: July 28, 2020). This study is conducted in accordance with GCP-ICH-6^[22]^ in a single university affiliated hospital (CHU de Besancon, Besancon, France). Eligible patients are screened and informed during the anesthesia consultation at least 2 days before surgery. Written informed consent is obtained by investigators prior to inclusion the day before surgery.

### 4.2. Planning and dissemination

Inclusions started on September 29, 2020. The initial planned duration of the trail was 24 months, but the recruitment was deeply impacted by the SARS-CoV2 pandemia. Protocol amendments for study prolongation have been approved by the French Ethics Committee and communicated to all investigators and trial registries. The university hospital of Besancon (CHU Besancon, Besancon, France) is the trial sponsor and the holder of all data and publication rights. The results of the study will be submitted for publication in a peer-review international medical journal and presented in abstract form in national and international conferences.

## Author contributions

Guillaume Besch: conception and study design; writing the manuscript; future analysis and interpretation of study data.

Anne-Laure Parmentier: conception and study design; critically reviewing the manuscript; future analysis of study data.

Francis Berthier: critically reviewing the manuscript; future analysis and interpretation of study data.

Helene Jaeg: critically reviewing the manuscript: future acquisition and interpretation of study data.

Julien Villeneuve: critically reviewing the manuscript: future acquisition and interpretation of study data.

Fethi Hammoudi: critically reviewing the manuscript: future acquisition and interpretation of study data.

Nans Scaringella: critically reviewing the manuscript: future acquisition and interpretation of study data.

Anne-Laure Clairet: critically reviewing the manuscript: future interpretation of data.

Lucie Vettoretti: critically reviewing the manuscript: future acquisition and interpretation of study data.

Gilles Chopard: conception and study design; critically reviewing the manuscript: future analysis and interpretation of study data.

Laurent Thines: critically reviewing the manuscript: future analysis and interpretation of study data.

David Ferreira: critically reviewing the manuscript: future analysis and interpretation of study data.

Emmanuel Samain: critically reviewing the manuscript: future analysis and interpretation of study data.

Sebastien Pili-Floury: conception and study design; critically reviewing the manuscript: future analysis and interpretation of study data.

All authors approved the final version of the manuscript to be published and are accountable for all aspects of the work.

**Conceptualization:** Guillaume Besch, Anne-Laure Parmentier, Gilles Chopard, and Sebastien Pili-Floury.

**Data curation:** Guillaume Besch, Sebastien Pili-Floury, and Emmanuel Samain.

**Formal analysis:** Guillaume Besch, Anne-Laure Parmentier, Francis Berthier, Helene Jaeg, Julien Villeneuve, Fethi Hammoudi, Nans Scaringella, Anne-Laure Clairet, Lucie Vettoretti, Gilles Chopard, Laurent Thines, David Ferreira, Emmanuel Samain, and Sebastien Pili-Floury.

**Funding acquisition:** Guillaume Besch and Sebastien Pili-Floury.

**Investigation:** Guillaume Besch, Francis Berthier, Helene Jaeg, Julien Villeneuve, Fethi Hammoudi, Nans Scaringella, Lucie Vettoretti, and Laurent Thines.

**Methodology:** Guillaume Besch, Anne-Laure Parmentier, Gilles Chopard, and Sebastien Pili-Floury.

**Project administration:** Guillaume Besch, Francis Berthier, Lucie Vettoretti, Laurent Thines, Emmanuel Samain, and Sebastien Pili-Floury.

**Resources:** Guillaume Besch, Sebastien Pili-Floury, and Lucie Vettoretti.

**Supervision:** Guillaume Besch, Francis Berthier, Laurent Thines, Emmanuel Samain, and Sebastien Pili-Floury.

**Validation:** Guillaume Besch, Anne-Laure Parmentier, Francis Berthier, Gilles Chopard, Laurent Thines, David Ferreira, and Emmanuel Samain Sebastien Pili-Floury.

**Visualization:** Guillaume Besch, Lucie Vettoretti, Francis Berthier, Helene Jaeg, Julien Villeneuve, Fethi Hammoudi, Nans Scaringella, Laurent Thines, David Ferreira, Emmanuel Samain, and Sebastien Pili-Floury.

**Writing – original draft:** Guillaume Besch.

**Writing – review and editing:** Guillaume Besch, Anne-Laure Parmentier, Francis Berthier, Helene Jaeg, Julien Villeneuve, Fethi Hammoudi, Nans Scaringella, Anne-Laure Clairet, Lucie Vettoretti, Gilles Chopard, Laurent Thines, David Ferreira, Emmanuel Samain, and Sebastien Pili-Floury.
